# Chemotherapy May Influence Esophageal Lugol Chromoendoscopy Severity: A Retrospective Cohort Study and Literature Review

**DOI:** 10.1002/kjm2.70020

**Published:** 2025-04-29

**Authors:** Wen‐Hung Hsu, Chien‐Chieh Lin, Hsiang‐Yao Shih, Leong‐Perng Chan, Hui‐Ching Wang, Yi‐Hsun Chen, Yu‐Chung Hsu, Hui‐Min Hsieh, I‐Chen Wu

**Affiliations:** ^1^ Division of Gastroenterology, Department of Internal Medicine Kaohsiung Medical University Hospital Kaohsiung Taiwan; ^2^ Division of Gastroenterology, Department of Internal Medicine Kaohsiung Medical University Gangshan Hospital Kaohsiung Taiwan; ^3^ Department of Public Health Kaohsiung Medical University Kaohsiung Taiwan; ^4^ College of Medicine Kaohsiung Medical University Kaohsiung Taiwan; ^5^ Department of Otolaryngology‐Head and Neck Surgery Kaohsiung Medical University Hospital Kaohsiung Taiwan; ^6^ Division of Hematology and Oncology, Department of Internal Medicine Kaohsiung Medical University Hospital Kaohsiung Taiwan; ^7^ Department of Pathology Kaohsiung Medical University Hospital, Kaohsiung Medical University Kaohsiung Taiwan; ^8^ Biomedical Artificial Intelligence Academy Kaohsiung Medical University Kaohsiung Taiwan

**Keywords:** chemotherapy, endoscopic submucosal dissection (ESD), esophageal squamous cell neoplasm (ESCN), Lugol chromoendoscopy

## Abstract

Lugol chromoendoscopy is widely used for screening esophageal squamous cell neoplasms (ESCNs) and evaluating dissection margins during endoscopic submucosal dissection (ESD). However, morphological variations may arise following treatment for synchronous cancers. This study aimed to assess the impact of chemotherapy on Lugol chromoendoscopy findings during both screening and ESD. From August 2009 to March 2024, ESCN patients undergoing esophageal ESD were enrolled. Lugol chromoendoscopy findings were analyzed based on four criteria: iodine staining, lesion size, margin assessment, and the pink‐color sign. Findings from screening endoscopy and ESD were compared, and medical records were reviewed for chemotherapy and radiation data. A literature review was also conducted to explore the potential effects of chemotherapy on Lugol chromoendoscopy findings. Among the 162 patients in this ESD cohort, 32 (19.8%) demonstrated notable differences between initial and follow‐up Lugol chromoendoscopy. Among them, 14 (43.8%) had undergone chemotherapy prior to ESD, while 26 received chemotherapy within 2 months before ESD. A significant proportion (53.8%) of chemotherapy‐treated patients exhibited faded chromoendoscopy findings (*p* < 0.01), despite no esophageal radiation exposure. Literature review findings supported our observation that chemotherapy may reduce Lugol voiding lesions. These findings suggest that chemotherapy influences lesion size and morphology during Lugol chromoendoscopy, underscoring the importance of careful timing and interpretation in these patients.

**Abbreviations**
CRT: chemoradiotherapy
ESCC: esophageal squamous cell carcinoma
ESCN: esophageal squamous cell neoplasm
ESD: Endoscopic submucosal dissection
HNSCC: head and neck squamous cell carcinoma
LVLs: Lugol‐voiding lesions
NBI: narrow band imaging

## 1 Introduction

Esophageal cancer ranks as the seventh leading cause of cancer‐related deaths worldwide [1]. The predominant histological type in Asia is esophageal squamous cell carcinoma (ESCC) [2, 3]. Over the past decade, endoscopic screening using image‐enhanced endoscopy has significantly improved outcomes for high‐risk populations, such as patients with head and neck squamous cell carcinoma (HNSCC) [4, 5]. Multidisciplinary approaches for aerodigestive squamous cell neoplasms are crucial for both HNSCC and ESCC. Chemotherapy, radiotherapy, endoscopic screening/treatment, and operation were all organized in such patient care, and all these modalities influence each other. Lugol chromoendoscopy, one of the earliest image‐enhanced endoscopy techniques for esophageal squamous cell neoplasms (ESCNs), selectively stains normal squamous epithelium brown, leaving dysplastic or cancerous areas unstained [6, 7]. This approach enhances the sensitivity of endoscopists in detecting early ESCNs.

While emerging virtual imaging techniques, such as narrow‐band imaging (NBI), [8, 9] have demonstrated comparable performance to Lugol chromoendoscopy, the latter remains the most widely utilized method in population‐level esophageal cancer screening programs [9, 10, 11]. Beyond its role in screening, Lugol chromoendoscopy is a well‐established method for evaluating dissection margins during endoscopic submucosal dissection (ESD) of early ESCNs [12, 13, 14]. However, variations in morphological findings during Lugol chromoendoscopy are frequently observed.

This study aims to investigate the influence of chemotherapy on variations in Lugol chromoendoscopy findings between screening endoscopy and endoscopic resection.

## 2 Methods

### 2.1 Esophageal Squamous Neoplasm Screening and Lugol Chromoendoscopy

**FIGURE 1:**
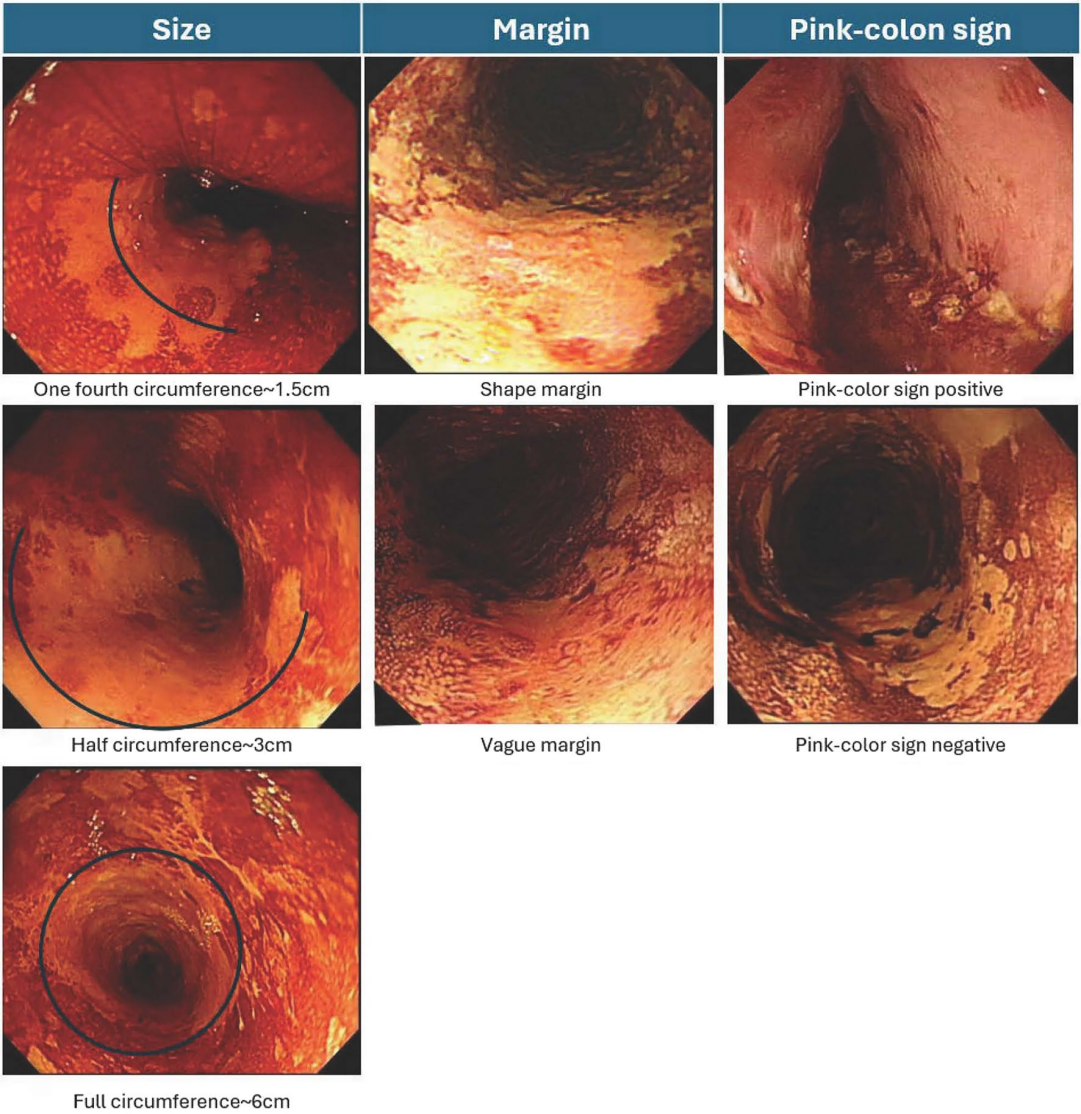
Illumination of variations in Lugol chromoendoscopy.

Since 2008, Kaohsiung Medical University Hospital has implemented upper endoscopy surveillance for high‐risk populations to detect ESCNs [15]. A 1%–3% Lugol solution spray chromoendoscopy is employed to identify superficial squamous lesions. Additionally, Lugol chromoendoscopy is used to evaluate lesion size prior to ESD for risk management, such as preventing delayed stricture formation. Findings from Lugol chromoendoscopy were recorded based on four criteria (Figure 1):

1. Background iodine staining: [6]

- A: No Lugol‐voiding lesions
- B: ≤ 10 small Lugol‐voiding lesions
- C: > 10 small Lugol‐voiding lesions
- D: Many irregularly shaped, multiform Lugol‐voiding lesions

2 Lesion margin: Sharp or vague.
3 Lesion size: Width and circumference were estimated (e.g., 6 cm for full circumference, 3 cm for half circumference, 1.5 cm for quarter circumference). Length was measured as the distance from the oral side to the anal side relative to the incisors.
4 Pink‐color sign: Presence or absence.

Targeted biopsies were performed for pathological confirmation.

### 2.2 Endoscopic Submucosal Dissection for Superficial Esophageal Neoplasms

Patients with superficial ESCNs deemed eligible for endoscopic resection underwent further evaluation with magnifying endoscopy or endoscopic ultrasound. The indication for esophageal ESD was based on the recommendations of the Japan Gastroenterological Endoscopy Society Guidelines [16]. All ESD procedures were performed by the first author, Dr. Hsu WH, under general anesthesia with endotracheal intubation to ensure airway protection. Vital signs were continuously monitored throughout the procedure. A 1%–3% Lugol chromoendoscopy was used to delineate the resection margins. Lesion characteristics recorded during ESD were consistent with those observed during the initial screening Lugol chromoendoscopy. The ESD procedure involved lesion marking, mucosal incision, and submucosal dissection following standard protocols. Resected specimens were sent for pathological examination.

### 2.3 Comparison of Lugol Chromoendoscopy Findings and Medical Record Review

Findings from screening Lugol chromoendoscopy and ESD Lugol chromoendoscopy were compared based on three parameters: lesion size, margin clarity, and the pink‐color sign as below. Dr. Hsu WH performed all of the 2nd chromoendoscopy and compared the findings without considering the treatment for other cancers when performing the procedures.

- Inconsistency in size: Defined as a variation in area (cm^2^) exceeding 25%.
- Inconsistency in margin: Defined as a change from sharp to vague or vice versa.
- Inconsistency in pink‐color sign: Defined as the disappearance of the pink‐color sign if it is initially positive during screening, or vice versa.

Lesions were considered dissimilar if two of the three parameters showed inconsistency. Retrospective medical record reviews were conducted to determine the date of the initial Lugol chromoendoscopy screening and the date of ESD. Records were also reviewed for systemic neoadjuvant treatments, including chemotherapy and radiation therapy, administered for aerodigestive squamous cell neoplasms. Chemotherapy agents used included fluorouracil, cisplatin, carboplatin, docetaxel, and cetuximab.

### 2.4 Eligibility Criteria for Literature Review on the Effects of Chemotherapy on Lugol Chromoendoscopy

A literature review was conducted to explore the impact of chemotherapy or chemoradiotherapy on Lugol‐voiding lesions (LVLs). Relevant studies were identified through an electronic search of PubMed (January 2010 to July 2024) using Medical Subject Headings (MeSH) terms such as “Lugol chromoendoscopy,” “chemotherapy,” “chemoradiotherapy,” and “narrow band imaging.” Studies focusing on changes in LVLs after systemic treatments, including variations in lesion size, margin clarity, and mucosal appearance, were selected for review.

## 3 Statistical Analysis

Demographic and clinical characteristics were summarized using descriptive statistics. Continuous variables were expressed as the mean and standard error and analyzed using the Mann–Whitney *U* test, while categorical variables were presented as counts and percentages and analyzed using the chi‐square test. A two‐tailed *p*‐value < 0.05 was considered statistically significant. All statistical analyses were conducted using STATA 15 software.

## 4 Results

### 4.1 Patient Characteristics and ESD Sessions

Between August 2009 and March 2024, 197 ESD sessions were performed for superficial ESCN among 162 patients. Of these, 35 sessions were excluded: 17 due to the absence of Lugol chromoendoscopy prior to ESD and 18 due to low‐quality Lugol chromoendoscopy images. This left 162 ESD sessions included in the analysis (Table 1).

**TABLE 1:** Clinical characteristics of patients with superficial ESCN treated with ESD (*n* = 162).

Characteristics	Value
Age (mean ± SE)	56.83 ± 0.67
Sex, male (%)	156 (96.3%)
Synchronous head and neck cancer, *N* (%)	124 (76.5%)
Lesion location, *N* (%)	
Upper esophagus	15 (9.3%)
Middle esophagus	94 (58.0%)
Lower esophagus	53 (32.7%)
Characteristics of Lugol chromoendoscopy, *N* (%)	
LVL classification	
A	1 (0.6%)
B	49 (30.3%)
C	83 (51.2%)
D	29 (17.9%)
Margin	
Sharp	139 (85.8%)
Vague	23 (14.2%)
Size (cm^2^)	
< 5	68 (42.0%)
5–10	41 (25.3%)
> 10	53 (32.7%)
Pink‐color sign	
Yes	124 (76.5%)
No	38 (23.5%)
ESD Pathology (depth of invasion), *N* (%)	
Low‐grade dysplasia	14 (8.6%)
High‐grade dysplasia	83 (51.2%)
T1a	39 (24.1%)
T1b	26 (16.1%)

Abbreviations: ESCN, esophageal squamous neoplasm; ESD, endoscopic submucosal dissection; LVL, lugol voiding lesion.

Among the 162 patients, 124 (76.5%) had synchronous head and neck squamous cell carcinoma. The majority of lesions (147/162, 90.7%) were located in the middle and lower esophagus. Lugol chromoendoscopy findings during ESD revealed that type C/D backgrounds predominated (112/162). Histopathological evaluation of ESD specimens showed that 91.4% (148/162) were classified as high‐grade squamous neoplasms.

### 4.2 Variations in Lugol Chromoendoscopy Findings

A paired analysis of screening Lugol chromoendoscopy and ESD Lugol chromoendoscopy revealed inconsistencies in 32 patients (19.8%) (Table 2). Among those, 23 (71.9%) had undergone prior chromotherapy, with 14 (43.8%) within 2 months. The inconsistent group had a higher grade of LVL classifications in screening Lugol chromoendoscopy compared to the consistent group (group C/D: 90.6% vs. 70.7%, *p* = 0.02). The inconsistent group displayed regression during ESD, characterized by smaller lesions (*p* = 0.04) with vague margins and disappearance of the pink‐color sign (both *p* < 0.01). Histopathological analysis further indicated less severe lesion grades in this group (*p* < 0.01). Chemotherapy exposure was a significant predictor for Inconsistent Lugol Chromoendoscopic findings (*p* < 0.01, Table 2). In the subgroup analysis, there were significant differences in consistency between the group without chemotherapy and the group with chemotherapy exposure within 2 months (*p* < 0.01), as well as between the group with chemotherapy exposure within 2 months and the group with exposure of 2 months or more (*p* < 0.01).

**TABLE 2:** Comparison between patients with consistent and inconsistent Lugol chromoendoscopic findings.

	Inconsistent (*n* = 32)	Consistent (*n* = 130)	*p*‐value
Age (mean ± SE)	55.28 ± 1.38	57.21 ± 0.76	0.25
Sex (male/female)	32 (100%)/0 (0%)	124 (95.4%)/6 (4.6%)	0.60
Synchronous head and neck cancer	30 (93.8%)	94 (72.3%)	< 0.01
Lesion Location (U/M/L)	5 (15.6%)/14 (43.8%)/13 (40.6%)	10 (7.7%)/80 (61.5%)/40 (30.8%)	0.13
Screening Lugol chromoendoscopy			
LVL classification (A/B/C/D)	0 (0%)/3 (9.4%)/12 (37.5%)/17 (53.1%)	1 (0.8%)/37 (28.5%)/57 (43.8%)/35 (26.9%)	0.02
Size (cm^2^) (< 5/5–10/ > 10)	11 (34.4%)/11 (34.4%)/10 (31.2%)	46 (35.4%)/38 (29.2%)/46 (35.4%)	0.84
Margin (sharp/vague)	25 (78.1%)/7 (21.9%)	115 (88.5%)/15 (11.5%)	0.15
Pink‐color sign (yes/no)	26 (81.3%)/6 (18.7%)	99 (76.2%)/31 (23.8%)	0.54
Biopsy pathology (LGD/HGD/SCC)	1 (3.1%)/24 (75.0%)/7 (21.9%)	2 (1.5%) /102 (78.5%)/26 (20.0%)	0.58
ESD Lugol chromoendoscopy			
LVL classification (A/B/C/D)	0 (0%)/8 (25.0%)/ 17 (53.1%)/7 (21.9%)	1 (0.8%)/41 (31.5%)/ 66 (50.8%)/22 (16.9%)	0.76
Size (cm^2^) (< 5/5–10/ > 10)	19 (59.4%) /8 (25.0%) /5 (15.6%)	49 (37.7%)/33 (25.4)/48 (36.9%)	0.04
Margin (sharp/vague)	19 (59.4%) /13 (40.6%)	120 (92.3%)/10 (7.7%)	< 0.01
Pink‐color sign (yes/no)	19 (59.4%)/13 (40.6%)	19 (14.6%)/111 (85.4%)	< 0.01
ESD pathology (LGD/HGD/T1a/T1b)	9 (28.1%)/16 (50.0%)/ 6 (18.8%)/1 (3.1%)	5 (3.9%)/67 (51.5%)/ 33 (25.4%)/25 (19.2%)	< 0.01
Interval between 1st & 2nd Lugol chromoendoscopy (days, mean ± SE)	48.31 ± 6.50	36.88 ± 2.50	0.06
Chemotherapy exposure			< 0.01
Never	9 (28.1%)	76 (58.5%)	
Excess 2 months	9 (28.1%)	42 (32.3%)	
Within 2 months	14 (43.8%)	12 (9.2%)	

Abbreviations: ESD, endoscopic submucosal dissection; HGD, high‐grade dysplasia; LGD, low‐grade dysplasia; LVL, lugol voiding lesion; SCC, squamous cell carcinoma.

Among the 162 patients, 85 (52.5%) had never undergone chemotherapy, while 26 (16.0%) and 51 (31.5%) had received concurrent chemotherapy within or more than 2 months prior to ESD, respectively (Table 3). The three groups had similar distributions in age, gender, and lesion site, but showed some differences in specific screening and follow‐up endoscopic findings, such as lesion size. Among those who had undergone chemotherapy, 73 patients had synchronous head and neck cancers treated with platinum plus 5‐fluorouracil, whereas one patient with colorectal cancer received FOLFOX (oxaliplatin and 5‐fluorouracil) within 2 months before ESD. The interval between two endoscopic exams was longest in patients who received chemotherapy within 2 months, followed by those who received no chemotherapy or had a delay of more than 2 months (54.4 vs. 33.6 and 26.5 days, respectively; *p* = 0.04). The inconsistency rates were 10.6%, 53.8%, and 17.6% among patients without chemotherapy, with chemotherapy within 2 months, and with chemotherapy more than 2 months prior to ESD, respectively (*p* < 0.01) (Table 3).

**TABLE 3:** Comparison of Lugol chromoendoscopic findings across 3 chemotherapy exposure groups.

	Without chemotherapy (*N* = 85)	Chemotherapy within 2 months (*N* = 26)	Chemotherapy excess 2 months (*N* = 51)	*p*‐value
Age (mean ± SE)	57.5 ± 0.87	55.12 ± 1.90	56.65 ± 1.23	0.47
Sex (male/female)	80 (94.1%)/5 (5.9%)	25 (96.2%)/1 (3.8%)	51 (100%)/0 (0%)	0.20
Synchronous head and neck cancer	51 (60.0%)	25 (96.2%)	48 (94.1%)	< 0.01
Lesion Location (U/M/L)	7 (8.2%)/48 (56.5%)/30 (35.3%)	2 (7.7%)/15 (57.7%)/9 (34.6%)	6 (11.8%)/31 (60.8%)/14 (27.4%)	0.88
Screening Lugol chromoendoscopy				
LVL classification (A/B/C/D)	1 (1.2%)/23 (27.1%)/30 (35.3%)/31 (36.5%)	0 (0%)/5 (19.2%)/10 (38.5%)/11 (42.3%)	0 (0%)/12 (23.5%)/29 (56.9%)/10 (19.6%)	0.13
Size (cm^2^) (< 5/5–10/ > 10)	25 (29.4%)/35 (41.2%)/25 (29.4%)	9 (34.6%)/4 (15.4%)/13 (50.0%)	23 (45.1%)/10 (19.6%)/18 (35.3%)	0.02
Margin (sharp/vague)	75 (88.2%)/10 (11.8%)	21 (80.8%)/5 (19.2%)	44 (86.3%)/7 (13.7%)	0.58
Pink‐color sign (yes/no)	64 (75.3%)/21 (24.7%)	20 (76.9%)/6 (23.1%)	41 (80.4%)/10 (19.6%)	0.79
Biopsy pathology (LGD/HGD/SCC)	1 (1.2%)/64 (75.3%)/20 (23.5%)	0 (0%)/22 (84.6%)/4 (15.4%)	2 (3.9%)/40 (78.4%)/9 (17.7%)	0.63
ESD Lugol chromoendoscopy				
LVL classification (A/B/C/D)	1 (1.2%)/24 (28.2%)/41 (48.2%)/19 (22.4%)	0 (0%)/8 (30.8%)/14 (53.8%)/4 (15.4%)	0 (0%)/17 (33.3%)/28 (54.9%)/6 (11.8%)	0.75
Size (cm^2^) (< 5/5–10/ > 10)	26 (30.6%)/30 (35.3%)/29 (34.1%)	14 (53.8%)/3 (11.5%)/9 (34.6%)	28 (54.9%)/8 (15.7%)/15 (29.4%)	0.01
Margin (sharp/vague)	75 (88.2%)/10 (11.8%)	20 (76.9%) /6 (23.1%)	44 (86.3%)/7 (13.7%)	0.35
Pink‐color sign (yes/no)	67 (78.8%)/18 (21.2%)	14 (53.9%)/12 (46.2%)	43 (84.3%)/8 (15.7%)	< 0.01
ESD pathology (LGD/HGD/T1a/T1b)	3 (3.5%)/39 (45.9%)/25 (29.4%)/18 (21.2%)	4 (15.4%)/15 (57.7%)/4 (15.4%)/3 (11.5%)	7 (13.7%)/29 (56.9%)/10 (19.6%)/5 (9.8%)	0.06
Interval between 1st & 2nd Lugol Chromoendoscopy (days, mean ± SE)	33.6 ± 3.3	54.4 ± 7.9	26.3 ± 3.9	0.04
Consistent (yes/no)	76 (89.4%)/9 (10.6%)	12 (46.2%)/14 (53.8%)	42 (82.4%)/9 (17.6%)	< 0.01

Abbreviations: ESD, endoscopic submucosal dissection; HGD, high‐grade dysplasia; LGD, low‐grade dysplasia; LVL, lugol voiding lesion; SCC, squamous cell carcinoma.

**FIGURE 2:**
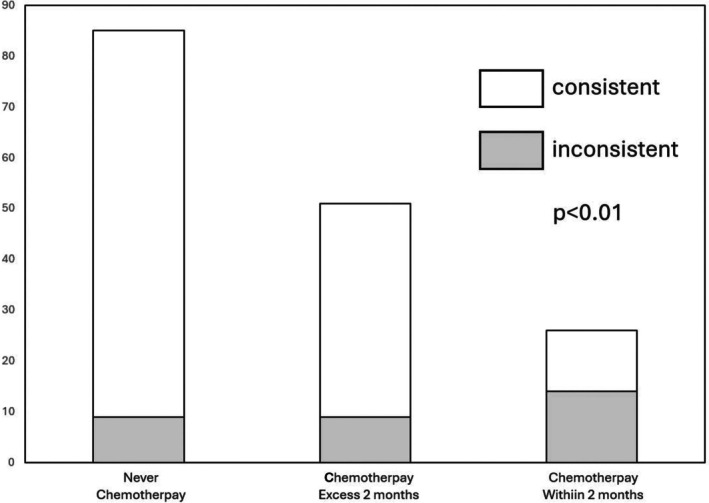
Influence of chemotherapy in Lugol chromoendoscopy. Recent chemotherapy caused a higher portion of inconsistencies in esophageal Lugol chromoendoscopy (*p* < 0.01).

Compared to patients who did not receive chemotherapy, the chemotherapy group exhibited significantly more cases of faded Lugol chromoendoscopy findings, with 53.8% (14/26) showing regression (*p* < 0.01) (Figure 2). Histopathological examination of ESD specimens in this group revealed notable degeneration of dysplasia when compared to initial screening biopsy results. Among the 26 patients who received chemotherapy within 2 months prior to ESD, those with inconsistent findings (*N* = 12) had higher LVL classification (grade C/D 100% vs. 58.3%, *p* < 0.01) in screening endoscopy and a longer interval between screening and pre‐ESD endoscopic exams (54.4 vs. 26.3 days; *p* < 0.01) (Supplementary Table 1).

**FIGURE 3:**
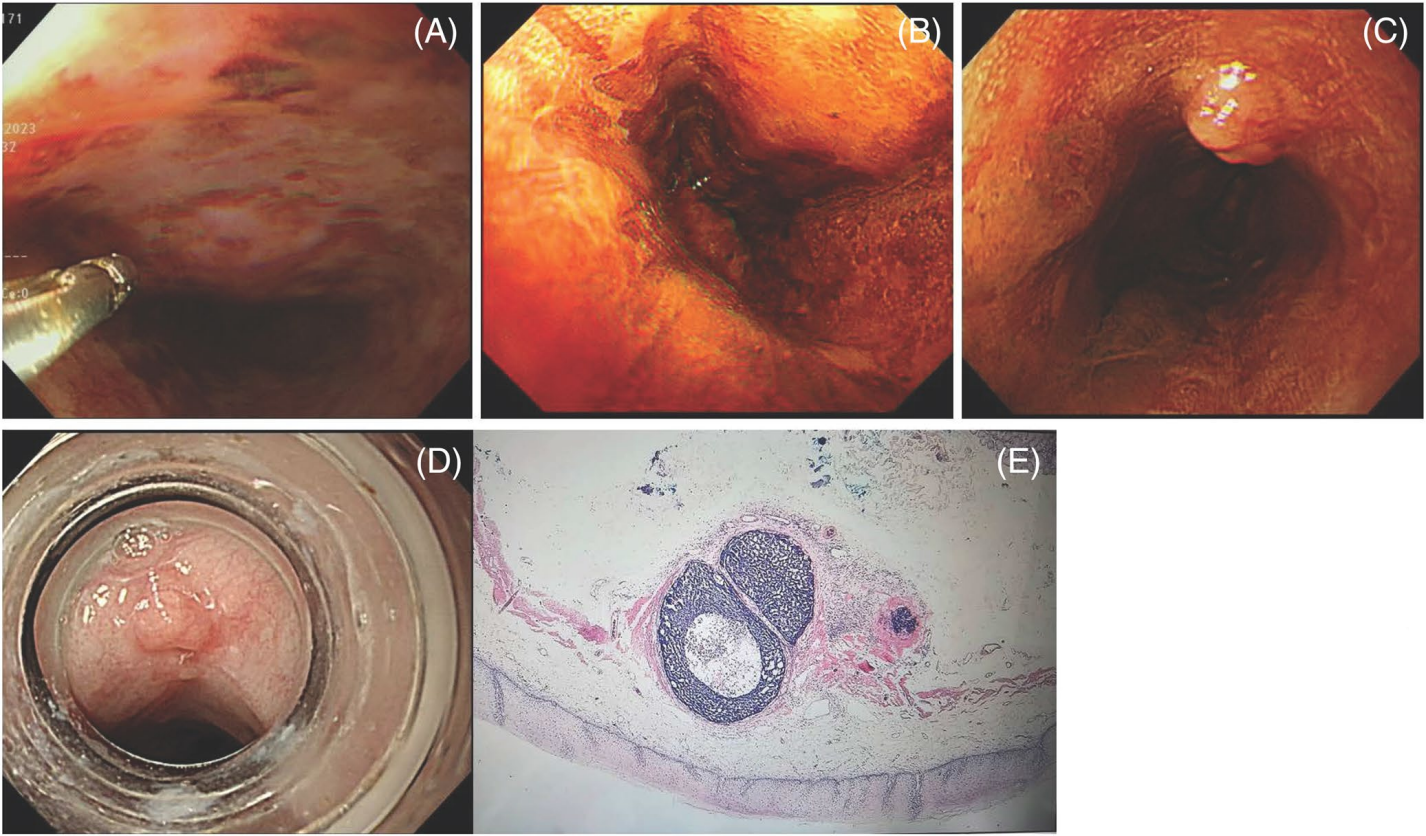
Screening with Lugol chromoendoscopy revealed a half‐circumferential esophageal LVL with a positive pink‐color sign (A). The patient received induction chemotherapy for tonsil cancer, and follow‐up Lugol chromoendoscopy 2 months later showed that the margin had become vague and the pink‐color sign had disappeared (B). Six months after the initial EGD, a follow‐up examination revealed an esophageal subepithelial lesion (C), which was removed by endoscopic mucosal resection with ligation (D). Histopathological examination (E) confirmed the presence of embedded squamous carcinoma.

Chemotherapy may affect lesion boundaries, raising concerns about the risk of incomplete endoscopic resection. For example, a 62‐year‐old male was newly diagnosed with tonsil squamous cell carcinoma, classified as cT4N1M0. Initial screening with Lugol chromoendoscopy revealed a half‐circumferential esophageal lesion with a positive pink‐color sign (Figure 3A). The patient underwent induction chemotherapy for tonsil cancer, consisting of docetaxel, cisplatin, and fluorouracil. Two months later, he underwent esophageal ESD, and follow‐up Lugol chromoendoscopy showed a vague margin with the disappearance of the pink‐color sign (Figure 3B). Six months after the initial endoscopy, a follow‐up examination revealed an esophageal subepithelial lesion (Figure 3C). This lesion was successfully removed by endoscopic mucosal resection with ligation (Figure 3D), and histopathological analysis confirmed the presence of embedded squamous carcinoma.

### 4.3 Findings From Literature Review

According to the review, we did not find any previous studies comparing esophageal endoscopic findings following chemotherapy or chemoradiotherapy (CRT) for synchronous cancers, particularly HNSCC. The literature review identified four Japanese studies that compared endoscopic findings before and after neoadjuvant chemotherapy or CRT for ESCC. These included one case series and three retrospective studies. Consistent with our findings, these studies reported significant regression in lesion size and changes in margin clarity following systemic chemotherapy or CRT (Table 4).

- Takahito Sugase et al. described a reduction in both size and number of LVLs post‐chemotherapy, though complete eradication of precancerous lesions was rare [17].
- Yoshiyuki Yukawa et al. observed reductions in size, number, and dysplasia grade of LVLs after chemotherapy [18].
- Suzuki et al. noted post‐CRT changes in esophageal lesions, describing an “irregular crack‐like pattern,” a feature frequently observed during our ESD assessments [19].
- Asada‐Hirayama et al. suggested that NBI with magnification may enhance the detection of subtle changes in LVLs following ESD and CRT, aligning with the altered endoscopic features we observed [20].

**TABLE 4:** Previous studies comparing Lugol chromoendoscopy findings after chemotherapy or CRT for ESCC.

Author, Year	Design	Disease treatment	Patients *N*	Baseline LVL	LVLs post chemo/CRT	Histopathology after chemo/CRT	Conclusion
Takahito Sugase et al. 2018 [17]	Case series	ESCC	5	Median diameter: 6.0 cm	1 patient: CR 3 patients: PR1 patient: PR (island‐like shape)	All showed residual cancer cells	LVLs reduction not equate to complete cancer eradication
Yoshiyuki Yukawa et al. 2012 [18]	Retrospective observational study	ESCC Neo‐Chemo	40	LVL group A: 6, B: 12, C: 9, D: 13	Group C and D: 77.3% of LVLs reduction. Group B: 33.3% of LVLs reduction	Improving from high‐grade dysplasia to lower‐grade or mild atypical lesions	Chemo could serve as a chemopreventive treatment for precancerous lesions
Suzuki et al. 2021 [19]	Retrospective observational study.	ESCC ESD ± CRT	146 ESD (CRT 64; control 82)	—	(1) Post CRT LVLs numbers: 58.1% reduction in multiple LVLs (2) Post CRT LVLs mucosal change: Irregular crack‐like pattern	Non‐significant	CRT may help prevent metachronous ESCC
Asada‐Hirayama et al. 2013 [20]	Retrospective clinical study	ESCC CRT after ESD	28 (72 lesions)	—	(1) PPV of LVL: 8.3%, *p* < 0.001 (2) NBI with Magnification: 85.7%	—	NBI with magnification could replace LVLs for surveillance in post‐CRT patients

Abbreviations: CR, complete remission; CRT, chemoradiotherapy; ESCC, esophageal squamous cell carcinoma; LVLs, Lugol voiding lesions; NBI, narrow band imaging; neo‐chemo, neoadjuvant chemotherapy; PR, partial remission.

## 5 Discussion

ESCNs are a significant concern in patients diagnosed with HNSCC [21]. Numerous studies, including ours, have documented the prevalence and risk factors for ESCNs in this population. However, reported incidence rates vary widely across studies. In our prospective screening of 815 incident HNSCC patients, 124 (15.2%) were diagnosed with ESCNs [15]. A European study using a similar methodology reported a synchronous esophageal neoplasia incidence of 21.9% (69/315) [22]. Another study of 1888 patients found a lower incidence of 7.9%, with most cases being superficial lesions [23]. A more recent and larger cohort study identified an even lower incidence of 4.5% among 13,627 HNSCC patients [5]. Variations in the timing and methodology of endoscopic screening likely explain these discrepancies. Our current study suggests that concurrent chemotherapy for HNSCC may also contribute to these differences by altering the visibility of esophageal lesions.

To the best of our knowledge, this is the first cohort study of superficial ESCNs to evaluate changes in Lugol chromoendoscopy findings following chemotherapy for advanced synchronous cancer without direct esophageal radiation exposure. Our findings highlight the dual impact of systemic chemotherapy on LVLs. Consistent with previous studies [17, 18] chemotherapy can induce regression in lesion size and clarity, potentially reducing the burden of high‐risk lesions. However, this regression complicates endoscopic evaluation by masking residual neoplasia, underscoring the limitations of Lugol chromoendoscopy post‐chemotherapy [19] Emerging imaging techniques, such as NBI with magnification, offer promise for enhancing the detection of subtle post‐treatment changes [20] Additionally, artificial intelligence‐assisted tools may further improve the differentiation of therapy‐induced changes from neoplastic lesions.

Early detection of ESCNs through screening significantly improves survival outcomes, as cancers identified at earlier stages generally have a better prognosis [5, 24]. ESD is a minimally invasive technique that enables precise removal of lesions confined to the mucosa and submucosa, offering curative potential without the need for extensive surgery [25, 26]. Patients with synchronous HNSCC and superficial ESCNs who undergo ESD experience significantly improved overall survival compared to those who do not receive treatment for their esophageal lesions. This highlights the importance of endoscopic resection as a viable management option for superficial ESCNs in this population, with no significant differences in treatment‐related morbidity or mortality compared to non‐treatment groups [27, 28].

Lugol chromoendoscopy remains a cost‐effective and widely accepted method for enhancing the visualization of esophageal mucosal lesions, particularly for detecting early‐stage cancers and dysplasia. Additionally, it plays a critical role in delineating lesion margins during ESD procedures. However, our findings, supported by previous studies, indicate that chemotherapy can induce changes in the esophageal mucosa and dysplastic lesions, which can alter Lugol staining patterns and complicate the interpretation of chromoendoscopic findings. In the group that underwent chemotherapy within 2 months prior to ESD, we observed a significant change in Lugol staining patterns among subjects with a longer interval between endoscopic exams. However, additional chemotherapy cycles may not be the sole contributing factor. Therefore, our data do not provide sufficient evidence to recommend performing ESD immediately after diagnosing an esophageal lesion. The optimal treatment sequence for patients with synchronous esophageal lesions and HNSCC remains unclear and should be determined on a case‐by‐case basis.

In real‐world clinical practice, severe HNSCC cases often take priority for treatment, leading to delays in endoscopic resection for asymptomatic early esophageal neoplasms until after chemotherapy. Our study suggests that such delays can result in altered Lugol chromoendoscopy findings, potentially accompanied by pathological changes. These alterations may affect lesion boundaries, raising concerns about the risk of incomplete endoscopic resection. Further research is needed to determine whether these changes correlate with increased risks of residual or recurrent neoplasia. Clinicians should be mindful of these potential changes and adjust the timing of Lugol chromoendoscopy and endoscopic resection to optimize outcomes for patients with synchronous cancers.

This study has several limitations. First, it was a single‐center, retrospective study with a relatively small sample size, introducing potential selection bias. Second, procedural differences may have influenced Lugol chromoendoscopy findings; most screenings were performed without sedation, whereas ESD procedures were conducted under sedation, potentially affecting staining time and image interpretation. Third, semi‐qualitative assessments, such as the presence or absence of the pink‐color sign, may be subject to intra‐observer variability. Moreover, since this was a retrospective study focused on superficial early esophageal cancer eligible for ESD as a curative treatment, it was difficult to generalize the findings to more advanced ESCC patients, whose lesions might be down‐staged after chemotherapy to become amenable to rescue ESD.

In conclusion, concurrent chemotherapy can significantly influence the morphology observed during Lugol chromoendoscopy. These findings emphasize the need for careful timing and interpretation of Lugol chromoendoscopy in patients undergoing chemotherapy for synchronous cancers. Further research is warranted to refine strategies for managing ESCNs in this clinical context.

## Ethics Statement

This study followed the Declaration of Helsinki and was approved by the Institutional Review Board of Kaohsiung Medical University Hospital (KMUHIRB‐E(I)‐20240308).

## Conflicts of Interest

The authors declare no conflicts of interest.

## Supporting information


**Supplementary Table 1.** Comparison between patients with consistent and inconsistent Lugol Chromoendoscopic findings after chemotherapy within 2 months prior to ESD (*N* = 26).

## Data Availability

All data generated or analyzed during the study are included in the published paper.
