# Supplementary material for: Chemotherapy May Influence Esophageal Lugol Chromoendoscopy Severity: A Retrospective Cohort Study and Literature Review
Source: Kaohsiung J Med Sci. 2025 Apr 29;41(7):e70020. doi: 10.1002/kjm2.70020 (PMC12407337; doi:10.1002/kjm2.70020)
Supplement: Supplementary file 1 — Supplementary Table 1. Comparison between patients with consistent and inconsistent Lugol Chromoendoscopic findings after chemotherapy within 2 months prior to ESD (N = 26). [file KJM2-41-e70020-s001.docx]

Supplementary Table 1. Comparison between patients with consistent and inconsistent Lugol Chromoendoscopic findings after chemotherapy within 2 months prior to ESD (N=26).

|  | Inconsistent  n=14 | Consistent  n=12 | P value |
| --- | --- | --- | --- |
| Age (mean±SE) | 51.7 ± 2.0 | 59.1 ± 3.1 | 0.05 |
| Sex (male/female) | 14 (100%)/0 (0%) | 11 (91.7%)/1 (8.3%) | 0.46 |
| Synchronous head and neck cancer | 14 (100%) | 11 (91.7%) | 0.46 |
| Lesion Location (U/M/L) | 2 (14.3%)/8 (57.1%)  /4 (28.6%) | 0 (0%)/7 (58.3%)  /5 (41.7%) | 0.59 |
| Screening Lugol chromoendoscopy |  |  |  |
| LVL classification (B/C/D) | 0 (0%)/4 (28.6%)  /10 (71.4%) | 5 (41.7%)/6 (50.0%)  /1 (8.3%) | <0.01 |
| Size (cm^2^) (<5/5-10/>10) | 3 (21.4%)/4 (28.6%)  /7 (50.0%) | 6 (50.0%)/0 (0%)  /6 (50.0%) | 0.11 |
| Margin (sharp/vague) | 10 (71.4%)/4 (28.6%) | 11 (91.7%)/1 (8.3%) | 0.33 |
| Pink-color sign (yes/no) | 12 (85.7%)/2 (14.3%) | 8 (66.7%)/4 (33.3%) | 0.37 |
| Biopsy pathology (HGD/SCC) | 11 (78.6%)/3 (21.4%) | 11 (91.7%)/1 (8.3%) | 0.60 |
| ESD Lugol chromoendoscopy |  |  |  |
| LVL classification (B/C/D) | 4 (28.6%)/6 (42.9%)  /4 (28.6%) | 4 (33.3%)/8 (66.7%)  /0 (0%) | 0.15 |
| Size (cm^2^) (<5/5-10/>10) | 8 (57.1%) /3 (21.4%)  /3 (21.4%) | 6 (50.0%)/0 (0%)  /6 (50.0%) | 0.22 |
| Margin (sharp/vague) | 8 (57.1%) /6 (42.9%) | 12 (100%)/0 (0%) | 0.02 |
| Pink-color sign (yes/no) | 3 (21.4%)/11 (78.6%) | 11 (91.7%)/1 (8.3%) | <0.01 |
| ESD pathology (LGD/HGD/T1a/T1b) | 4 (28.6%)/7 (50.0%)/  2 (14.3%)/1 (7.1%) | 0 (0%)/8 (66.6%)/  2 (16.7%)/2 (16.7%) | 0.27 |
| Interval between 1^st^ & 2^nd^ Lugol Chromoendosocpy (days, mean±SE) | 54.4 ± 7.9 | 26.3 ± 3.9 | <0.01 |
| Abbreviations: ESD: Endoscopic submucosal dissection; LGD: low grade dysplasia; HGD: high grade dysplasia; SCC: squamous cell carcinoma; LVL: lugol voiding lesion | | | |
